# Medical Mistrust Among Black Patients with Serious Illness: A Mixed Methods Study

**DOI:** 10.1007/s11606-024-08997-z

**Published:** 2024-08-26

**Authors:** Kristine L. Cueva, Arisa R. Marshall, Cyndy R. Snyder, Bessie A. Young, Crystal E. Brown

**Affiliations:** 1 Department of Medicine, University of Washington, Seattle, USA; 2 Division of Pulmonary, Critical Care, and Sleep Medicine, Department of Medicine, University of Washington, Seattle, WA USA; 3 Department of Family Medicine, Center for Health Workforce Studies, School of Medicine, University of Washington, Seattle, WA USA; 4 Division of Nephrology, Department of Medicine, University of Washington, Seattle, WA USA; 5 UW Justice, Equity, and Inclusion Center for Transformational Research, Office of Healthcare Equity, UW Medicine, University of Washington, Seattle, WA USA; 6 Cambia Palliative Care Center of Excellence at UW Medicine, Seattle, WA USA; 7 Department of Bioethics and Humanities, School of Medicine, University of Washington, Seattle, WA USA

**Keywords:** Racism, Discrimination, Medical mistrust, End of life, Serious illness

## Abstract

**Background:**

Medical mistrust among Black patients has been used to explain the existence of well-documented racial inequities at the end of life that negatively impact this group. However, there are few studies that describe patient perspectives around the impact of racism and discriminatory experiences on mistrust within the context of serious illness.

**Objective:**

To better characterize experiences of racism and discrimination among patients with serious illness and its association with medical mistrust.

**Participants:**

Seventy-two Black participants with serious illness hospitalized at an academic county hospital.

**Approach:**

This is a convergent mixed methods study using data from participant-completed surveys and existing semi-structured interviews eliciting participants’ perspectives around their experiences with medical racism, communication, and decision-making.

**Main Measures:**

The experience of medical racism and its association with Group-Based Medical Mistrust (GBMM) scale scores, a validated measure of medical mistrust.

**Key Results:**

Of the 72 Black participants, 35% participated in interviews. Participants were mostly men who had significant socioeconomic disadvantage, including low levels of wealth, income, and educational attainment. There were reported high levels of race-based mistrust in the overall GBMM scale score (mean [SD], 36.6 [9.9]), as well as high scores within the suspicion (14.2 [5.0]), group disparities in healthcare (9.9 [2.8]), and lack of support (9.1 [2.7]) subscales. Three qualitative themes aligned with the GBMM subscales. Participants expressed skepticism of healthcare workers (HCWs) and modern medicine, recounted personal experiences of discrimination in the medical setting, and were frustrated with poor communication from HCWs.

**Conclusions:**

This study found high levels of mistrust among Black patients with serious illness. Suspicion of HCWs, disparities in healthcare by race, and a lack of support from HCWs were overarching themes that influenced medical mistrust. Critical, race-conscious approaches are needed to create strategies and frameworks to improve the trustworthiness of healthcare institutions and workers.

**Graphical abstract:**

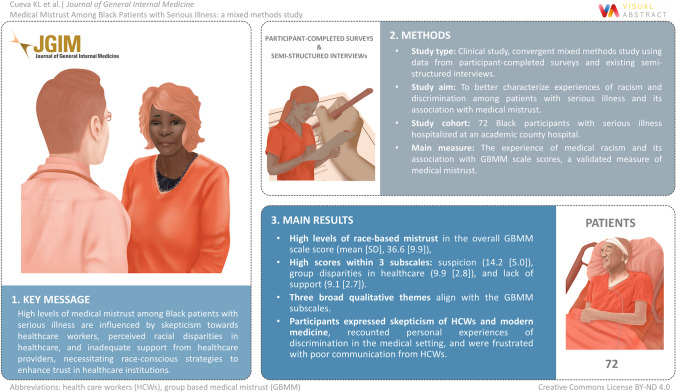

## INTRODUCTION

Racial inequities at end of life (EOL) are well-documented with Black patients more likely to receive higher intensity care and die in the hospital resulting in worse quality of life and higher healthcare costs compared to their White counterparts.^1–3^ Multiple patient-level factors, including mistrust in or suspicion of healthcare systems, have been used to explain these differences.^2,4,5^ Medical mistrust is also related to systemic racism and includes skepticism of health systems and healthcare workers (HCWs).^6–9^ This skepticism may extend further into fear of healthcare interactions and concerns about experimentation. For example, *Black iatrophobia*, described by Harriet A. Washington, is the fear of medicine in Black communities as a result of cruel, involuntary, and unethical treatment of Black people by HCWs and government intuitions throughout history.^10^ Indeed, medical and research injustices against Black and other racially minoritized groups are often cited as a reason for medical mistrust in Black communities.^11^ However, medical mistrust may also be a result of the poor quality of care that Black patients disproportionately experience in the modern day. For example, undertreated pain, poor quality of communication, and implicit biases associated with substandard care are all inequities currently experienced by Black patients.^12^ As a result, medical mistrust may be associated with decreased concordance with provider recommendations, appointment attendance, and an increased likelihood of poor self-reported health.^13–15^ High levels of medical mistrust among Black patients have also been associated with low levels of medication concordance and frequent discriminatory experiences in the healthcare setting.^16–18^ Within the context of serious illness, medical mistrust is associated with low serious illness preparedness and advanced care planning among Black patients.^19,20^

We recently reported the results of a qualitative study of Black patients with serious illness.^12^ In that study, participants reported experiencing discrimination from HCWs that negatively impacted their perspectives on patient-clinician communication and decision-making, and exacerbated medical mistrust. While medical mistrust among Black patients has been well-described, there are few studies using qualitative data describing Black patients’ perspectives around the impact of racism and discriminatory experiences on mistrust within the context of serious illness.^6,17,18^ In this paper, we report the results of a mixed methods study consisting of secondary qualitative analysis and a validated measure of medical mistrust to better characterize patient-reported experiences of racism and discrimination, its effects on their perceived care in the medical setting, and its associations with medical mistrust.

## METHODS

### Study Design

This is a convergent mixed methods study using a validated measure of medical mistrust and existing semi-structured interviews.^21^ The study was approved by the University of Washington Institutional Review Board (STUDY00011422).

### Participants

We recruited Black patients with serious illness hospitalized at an academic county hospital in Seattle, WA. Patients were screened using the electronic medical record (EMR) to identify potential participants at least 18 years of age, without cognitive dysfunction, and with a serious illness defined as a diagnosis associated with a median life expectancy of 2 years or less.^22^ Potential participants were approached in-person on hospital day 2 or 3 for enrollment in a prospective study examining racism and communication. While the EMR was used to identify potential participants, only patients who self-identified as Black were approached for an interview. After providing informed consent, participants completed a survey containing demographic information including self-reported race, health literacy, experiences with discrimination, and medical mistrust. Health literacy was measured by the Rapid Estimate of Adult Literacy in Medicine—Short Form (REALM-SF).^23^ Discrimination and medical mistrust were measured using the Discrimination in Medical Setting (DMS) scale and Group-Based Medical Mistrust (GBMM) scale, respectively.^7,24^ Surveys were completed by participants themselves or verbally if they were unable to write or visually impaired. After completing the surveys, participants were purposefully selected based on diagnosis, gender, and whether they endorsed experiencing discrimination on any of the DMS items to participate in an interview to gather more in-depth perspectives on their experiences. Informed consent for the interview was obtained either through writing or verbally if a patient was unable to write or visually impaired. Interviews occurred at bedside or by phone, depending on participant preference. A $20 gift card was provided for participations for both enrollment in the prospective cohort and the interview.

### Medical Mistrust Survey

The GBMM scale is a 12-item scale measuring race-based medical mistrust on a 5-point Likert scale, with 1 being “strongly disagree” and 5 being “strongly agree.” It is scored by the sum of responses with higher scores indicating more mistrust. Notably, survey items 2, 8, 10, and 11 are reverse-scored. The scale has three subscales: suspicion, group disparities in healthcare, and lack of support. The GBMM and its subscales have good construct validity, high internal consistency, and good convergent and discriminant validity among Black patients.^25–27^ High scores are associated with low cancer screening rates,^7^ poor medication and care concordance,^17,28^ hesitancy to seek addiction treatment,^29^ and low COVID-19 vaccination rates.^30^

### Interviews

We used existing qualitative data that were obtained concurrently with survey data from 25 Black patients in our cohort on how experiences with medical racism affect patient-clinician communication and decision-making.^12^ The interview guide was designed and refined to elicit perspectives around experiences with medical racism, communication, and decision-making to bring a critical, race-conscious approach to the research.^31–34^ During interviews, patients were asked open-ended questions on their experiences with processing and coping with racism and its impact on their healthcare experiences. They were asked to discuss how their experiences impacted their communication and decision-making as they neared EOL and to elaborate on survey responses. Interviews were 30–70 min in length and were recorded, deidentified, and transcribed. Qualitative analyses were conducted iteratively as interviews were ongoing. Interviews were conducted past the point of thematic saturation, where no new themes or codes were identified during analyses, for the purposes of the original research question.^35^

### Analysis

The methods for the original qualitative analyses have been described elsewhere.^12^ Briefly, CEB, a Black and Korean physician, and ARM, a multiracial, Asian and Pacific Islander research coordinator, conducted all interviews. CEB, ARM, CRS, a multiracial Black qualitative researcher, and CCP, a multiracial Hispanic research coordinator, reviewed the transcripts and, using open, inductive coding, produced a codebook. Preliminary themes and codes were reviewed, edited, and approved by the research team including KLC, a Filipina American physician. CEB, ARM, KLC, and CCP independently coded an initial set of three transcripts before meeting to identify and reconcile discrepancies by refining existing or defining new codes for a final codebook. CEB, ARM, KLC, and CCP coded the remaining transcripts with over 60% undergoing a secondary review.

For the analysis in this study, CEB and KLC reviewed excerpts within the transcripts that were coded within the theme of mistrust. Fresh transcripts were re-read by CEB and KLC to identify additional important excerpts or contextual features within the transcripts that provided additional description of participants’ mistrust. Codes and their accompanying excerpts were deductively re-coded and organized based on the GBMM subscales. These deductive codes and organization of the excerpts were reviewed and any coding disagreements were discussed and resolved. As the GBMM scale consists of a total of 12 items within the three subscales, no novel themes or codes were identified. Means and standard deviations of overall GBMM scores, subscales, and individual survey items of all participants were calculated. The interview and survey data were subsequently merged and organized in a joint display using a table to identify new insights beyond the information gained from the separate survey and interview results. The data were examined to evaluate the fit of the survey and interview data, confirming that the survey and interview data provided similar conclusions and were coherent.^36^ Dedoose was used to support all qualitative analyses.^37^ Stata/SE was used to support quantitative analyses.^38^

## RESULTS

Table 1 summarizes the demographics of the survey and interview respondents. Seventy-two Black participants completed the survey, of which 35% participated in interviews. Overall, participants were mostly men with significant socioeconomic disadvantage, including low levels of wealth, income, and educational attainment. Participants reported high levels of race-based mistrust in the overall GBMM scale (mean [SD], 36.6 [9.9]). Participants also demonstrated high scores within the suspicion (14.2 [5.0]), group disparities in healthcare (9.9 [2.8]), and lack of support (9.1 [2.7]) subscales (Table 2). Three broad qualitative themes aligned with the GBMM subscales (Table 3).

**Table 1:** Participant Demographics

	Cohort		Interview	
Variable	*N*	Statistic	*N*	Statistic
Age (years)^a^	72	61.0 (10.7)	25	62.0 (10.3)
Female^b^		22 (31.0)		5 (20.0)
Years of schooling^a^	69	13.0 (2.3)	24	13.4 (2.7)
Income^b^	68		24	
< $25,000		51 (75.0)		19 (79.2)
$25,000–34,999		6 (8.8)		2 (8.3)
$35,000–49,999		3 (4.4)		1 (4.7)
$50,000–74,999		3 (4.4)		2 (8.3)
$75,000–99,999		4 (5.9)		0
$100,000–149,999		1 (1.5)		0
Insurance^b,c^	72		25	
Medicaid		46 (63.9)		9 (36.0)
Medicare		27 (37.5)		6 (24.0)
Private		10 (13.9)		8 (32.0)
Uninsured		3 (4.2)		1 (4.0)
Other		1 (1.4)		1 (4.0)
Wealth^b^	64		25	
0		27 (39.1)		10 (40.0)
1		15 (21.7)		4 (16.0)
2		13 (18.8)		8 (32.0)
3		7 (10.1)		1 (4.0)
4		5 (7.3)		2 (8.0)
5		2 (2.9)		0
REALM-SF (health literacy)^a^	48	5.5 (2.2)	19	5.8 (2.0)
Comorbidities^b^	72		25	
NYHA class III or IV heart failure		29 (40.3)		13 (52.0)
Charlson ≥ 6		29 (40.3)		8 (32.0)
Metastatic cancer		9 (12.5)		2 (8.0)
Child’s class C cirrhosis or MELD > 17		2 (2.8)		0
ESRD and DM or albumin < 2.5		2 (2.8)		1 (4.0)
COPD FEV1 < 35% or O_2_-dependent		2 (2.8)		1 (4.0)
Age 75 or older^d^		1 (1.4)		1 (4.0)
Hospitalized^e^		1 (1.4)		0

^a^Mean (standard deviation); ^b^number (percentage); ^c^some patients held multiple types of insurances. *REALM-SF*, Rapid Estimate of Adult Literacy in Medicine—Short Form; *NYHA*, New York Heart Association; *MELD*, Model for End-Stage Liver Disease; *ESRD*, end-stage renal disease; *DM*, diabetes mellitus; *COPD*, chronic obstructive pulmonary disease; *FEV1*, forced expiratory volume in 1 s; *O*_*2*_, oxygen; *d*, age 75 or older with an existing condition associated with median survival of 2 years or less but of lesser severity; *e*, hospitalized from any case within the past 18 months with an existing condition associated with median survival of 2 years or less but of lesser severity

Wealth is measured by the presence of five assets (checking account, savings account, retirement funds, vehicle, and home ownership), and scored from 0 to 5.^53^ The REALM-SF is a measure of health literacy where participants are given 7 medical words to read aloud and is scored by the number of words correctly read^23^

**Table 2:** Group-Based Medical Mistrust Scores

	Cohort (*n* = 72)
Item	Mean (SD)
Total GBMM Score	36.6 (9.9)
Suspicion subscale	14.2 (5.0)
3. People of my ethnic group should not confide in doctors and HCWs because it will be used against them	2.7 (1.2)
4. People of my ethnic group should be suspicious of information from doctors and HCWs	2.9 (1.2)
5. People of my ethnic group cannot trust doctors and HCWs	2.7 (1.2)
6. People of my ethnic group should be suspicious of modern medicine	2.9 (1.2)
7. Doctors and HCWs treat people of my ethnic group like “guinea pigs”	3.0 (1.3)
9. Doctors and HCWs do not take the medical complaints of people of my ethnic group seriously	3.4 (1.2)
Group Disparities in Healthcare subscale	9.9 (2.8)
8. People of my group receive the same medical care from doctors and HCWs as people from other groups	3.2 (1.1)
10. People of my ethnic group are treated the same as people of other groups by doctors and HCWs	3.4 (1.1)
11. In most hospitals, people of different ethnic groups receive the same kind of care	3.3 (1.1)
Lack of Support subscale	9.1 (2.7)
1. Doctors and HCWs sometimes hide information from patients who belong to my ethnic group	3.3 (1.2)
2. Doctors have the best interests of people of my ethnic group in mind	2.8 (1.2)
12. I have personally been treated poorly or unfairly by doctors or HCWs because of my ethnicity	3.1 (1.4)

*Abbreviations*: *SD*, standard deviation; *HCW*, healthcare worker; 5-point Likert scale (1 = strongly disagree, 5 = strongly agree)

**Table 3:** Group-Based Medical Mistrust Survey Items and Representative Quotes

GBMM subscale	Survey item	Patient quote
Suspicion	3. People of my ethnic group should not confide in doctors and HCWs because it will be used against them	“[Speaking up] can make patients afraid of retaliation, maybe a patient dies.” “I’m not gonna say nothing. The last thing I’m gonna do is start [crap] with someone who’s going to decide whether I hurt or sleep well.”
	4. People of my ethnic group should be suspicious of information from doctors and HCWs	“There are times at the emergency room…they kind of go around the corner and they’re whispering and they keep turning around and looking at me. I can tell that they’re not being truthful about something, you know.”
	5. People of my ethnic group cannot trust doctors and HCWs	“I don’t trust these folks.” “I don’t trust [information from a biased doctor or HCW] at all. It makes me very skeptical and uneasy.”
	6. People of my ethnic group should be suspicious of modern medicine	“I don’t know what [the respiratory therapist] can do to that CPAP to hurt me or whatever but I’m saying, she’s just acting goofy. She went out of the room after she got me all hooked up and then she came back and did something to the machine. As soon as she left, I waited for about another hour and I took that thing off and unplugged that thing. I’m just paranoid now.” “Because I remember a time, my African American upbringing, we didn’t go to the emergency room, we didn’t go see a doctor. We, home remedies or whatever…like my brother. He died of colon cancer. And that could’ve been, and if he went in time, that could’ve been stopped. You see what I’m saying? You know?”
	7. Doctors and HCWs treat people of my ethnic group like “guinea pigs”	“I believe that a lot of us are given these diseases or incurring them and we’re just being treated for them. We’re not treated to be cured. We’re just ongoing medicated for money.”
	9. Doctors and HCWs do not take the medical complaints of people of my ethnic group seriously	“[The doctor] said, ‘We’re going to get you a wheelchair and get you on your way home.’ I said, ‘Home? Man, do you know what I just went through?’ Like that. I said, ‘I’m not going home. I don’t feel safe to go home.’” “[He said] My problem isn’t as serious as [another patient’s] or something. Something to that effect. I was very offended and hurt.”
Group Disparities in Healthcare	8. People of my group receive the same medical care from doctors or HCWs as people from other groups	“[HCWs] will have nothing to do with you if you are anything other than European. They will not touch you. They don’t care how much money you got, how much influence you got, you are a colored person, then you don’t count.”
	10. People of my ethnic group are treated the same as people of other groups by doctors and HCWs	“[Black and brown people are more likely to die in the hospital] because of who they are. Because the system is the one making them be ignored and not get sufficient treatment.”
	11. In most hospitals, people of different ethnic groups receive the same kind of care	“I don’t believe that people that are non-White get equal treatment.”
Lack of Support	1. Doctors and HCWs sometimes hide information from patients who belong to my ethnic group	“So Dr. XXX told me that I am going to put you back on [the transplant list], and then all these years later, that same doctor decided to go to the round table and call in my medical team and told them, ‘At this time she is no longer a candidate for transplant.’ So she just stabbed me in the back and didn’t say a thing.”
	2. Doctors have the best interests of people of my ethnic group in mind	“I believe some of [doctors and HCWs] are, like many other people, think it’s their job or are doing their best and there are some people that know and take advantage of these things and their privilege and they really don’t care, you know.”
	12. I have personally been treated poorly or unfairly by doctors or HCWs because of my ethnicity	“There are some people that assume that Black people are not capable of understanding what you’re saying or what you’re thinking or answering a question properly.”

*Abbreviations*: *GBMM*, Group-Based Medical Mistrust; *HCW*, healthcare worker

### Suspicion

Many participants expressed wariness and skepticism of HCWs and medicine (Table 3). Within the suspicion subscale, participants were most concerned about not having their concerns taken seriously (Item 9, 3.4 [1.2]). One participant shared, “There’s so much deceit that I’d be scared of what they do to me as far as surgeries and saying what’s wrong with me.” Another participant said, “Some, you can’t tell them anything. They’re doctors so they know everything. You may suggest something and they’ll try and make you feel stupid, saying ‘I’m a doctor, and I’m the only one that knows.’” Another recalled bringing up concerns their pain medications were not being increased because of their race: “There’s no response. Sometimes I hear, ‘Oh yes, that’s a very good point, I will look into it.’ But once they leave me in the bed, that’s the end of it.” Participants reported being worried they were treated like guinea pigs (Item 7, 3.0 [1.3]). One participant commented, “I feel that we can be victims of experimentation. People are devising ways to try new medications on people.”

Participants reported high levels of suspicion of modern medicine (Item 6, 2.9 [1.2]) and information given by HCWs (Item 4, 2.9 [1.2]). One participant stated, “There is medicine that I think are recommended to the market, to start being taken [by] patients which are not ready. They don’t stop the papers.” Another participant described his brother’s worry about his pacemaker: “[He] didn’t want to put up the radio because he had concerns that at some point somebody might decide to turn off his heart.” Some participants described skepticism over medical information delivered by HCWs: “I don’t trust the doctors at all. If it was anything serious, I would always want a second opinion.” Another participant described how he needed to do his own research to stay ahead of his doctors: “By telling [doctors] that you two steps behind, that’s letting them know that you don’t know what you need, and they can just string you along.”

### Group Disparities in Healthcare

Participants recounted multiple experiences in which there was a palpable inequity in how they were treated compared to patients from other racial backgrounds, particularly White patients. Multiple participants were troubled by this difference in treatment (Item 10, 3.4 [1.1]). One patient remarked, “I don’t trust [doctors] anymore because I felt like they showed me such disregard. If I was one of their White counterparts, they would’ve never did me like that.” Another talked about her experience in the intensive care unit where she observed a nurse was giving more attention to her neighbor because they were White and financially well off: “[The nurse] was catering and laughing over there and when I pushed the button for help, ‘I’ll be with you in a minute.’ Like I don’t matter. You putting on a show for those people because they got family and money, and I’m here by myself.”

Additionally, participants were disturbed by discrepancies in the care they received (Item 8, 3.2 [1.1]), noting that these differences were observed in most hospitals (Item 11, 3.3 [1.1]). A participant spoke about his experience when visiting the pain clinic: “They ask you questions, test your levels, all the stuff that they do to see if you’re actually taking the pills. I don’t believe they do that to White people.” Another participant recalled, “My daughter was having a baby and the anesthesiologist left her on the table to go give an epidural to the White lady in the next room.” Another participant described his cousin’s experience when seeking help for his anxiety: “They won’t give him anything because they think he’s just there for the drugs.” Overall, participants felt excluded from receiving appropriate medical care because of their race or ethnicity: “The medical community is very strong, and it has a lot of things that are helping people to survive death. But those are not applied to people of color.”

### Lack of Support

Many participants strongly felt that HCWs would sometimes hide information from them (Item 1, 3.3 [1.2]). One participant reported having to ask multiple questions to get answers from providers: “I feel like some people may not be getting the full truth.” She went on to say, “So that’s how come you have to ask the questions differently based on how the doctor approaches the situation.” Another participant described his frustration during a prior hospitalization: “That’s fifteen times you done drawn blood from me and you can’t tell what’s going on with me? Y’all been drawing blood from me, and I ain’t hear no results.”

In addition to withholding information from them, participants felt they were personally treated poorly by HCWs due to their race and ethnicity (Item 12, 3.1 [1.4]). One participant recalled overhearing a nurse describe a violent encounter with “Black gang members” to her co-workers, which impacted how she interacted with him: “And when it came time to treat me, she would always be in a rush, she would throw things at me, and if I didn’t take my medications right away, she would snatch them like a child from my tray and walk off with them.” Moreover, participants felt that doctors did not have their best interests in mind (Item 2, 2.8 [1.2]). One participant shared, “I’m not valued so your brain isn’t trying to figure out what you can do for me to possibly cure or heal me.”

## DISCUSSION

We described a mixed methods study showing high levels of medical mistrust among Black patients with serious illness. Themes around mistrust in our qualitative data align with the GBMM subscales—suspicion, group disparities in healthcare, and lack of support. Our results are consistent with prior studies showing high levels of medical mistrust among Black patients. However, participants in our study reported higher overall mean, subscale, and item scores compared to other studies investigating medical mistrust around COVID-19 vaccinations and addiction treatment among Black patients.^29,30^ We build upon our previous study by further expanding upon the theme of mistrust by merging survey and interview data to improve understanding of patients’ lived experiences.^6,12^ Our study adds to the existing literature by using qualitative data to amplify the voices of Black patients with serious illness and their perspectives, focusing on their views and experiences on medical mistrust.^6^

*Black iatrophobia* describes the fear of medicine in Black communities as the product of medical and research injustices against Black people throughout history.^10^ These atrocities, such as the Syphilis study conducted at Tuskegee and the sterilization of Black mothers and the resultant intergenerational trauma, are often cited as the cause for medical mistrust.^11,39–41^ Beyond these historical injustices and their lasting effects, experiences shared by our participants demonstrate that injustices continue to occur. Participants recounted personal and vicarious experiences in which they or others experienced disregard and dismissal by HCWs, lack of transparency in their care, and many other negative encounters resulting in a heightened suspicion of HCWs and modern medicine. This iatrophobia has been described in prior studies to play a role in adverse health consequences for Black people, including delays in preventative health screenings.^5,42^ For example, studies on low colorectal cancer screening rates among Black men described distress over the invasiveness of colonoscopy procedures and fear of experimentation,^42^ echoing the guardedness against medical services expressed by participants in our study.

Previous studies suggest that experiences with discrimination and bias in the healthcare setting are associated with medical mistrust, potentially impacting how Black patients with serious illness engage in their care, how prepared they feel to make decisions to address their serious illness, and care satisfaction.^20,43,44^ Medical mistrust is also cited as a barrier for Black patients with serious illness to participate in advanced care planning and hospice enrollment.^45,46^ However, studies often compare Black patients to their White counterparts, upholding White normativity as the standard. Additionally, existing traditional approaches to research on health disparities and medical mistrust are often rooted in a deficit-based lens.^47^ Deficit-based thinking is built on a foundation of racial and class bias by placing the onus of disparities upon patients, blaming them for having internal defects rather than investigating the inequitable societal structures and behaviors that produce these results.^48^ While multiple qualitative studies published in the past decade focus on medical mistrust among various populations,^49–51^ few have focused on Black patients with serious illness to provide insight on how their lived experiences impact medical mistrust as they approach EOL.^32^ Instead of using White populations as the standard comparison group, race-conscious approaches to research that prioritizes perspectives of Black patients may be one way to more meaningfully address mistrust and racialized experiences. Such strategies include improving provider diversity and opportunities for race-concordant interactions as well as creating spaces and communication frameworks that may improve therapeutic relationships between patients and HCWs.^52^

### Limitations

This study has some limitations. First, results may not be generalizable as the sample size was limited and patients enrolled were from a single academic county hospital. Second, the majority of eligible patients were men, resulting in Black women being underrepresented in this study despite efforts at targeted recruitment. Third, although participants in the cohort had a similar demographic distribution compared to those who participated in interviews, those who participated in interviews may have differing views from those who did not.

## CONCLUSION

In this mixed methods study, we describe high levels of medical mistrust among Black patients with serious illness. Disparities in healthcare by race, a lack of support from HCWs, and suspicion of HCWs and modern medicine are overarching themes that were associated with medical mistrust. This study highlights the need for additional critical, race-conscious approaches to generate strategies and frameworks to improve the trustworthiness of healthcare institutions, HCWs, and patient-clinician relationships.
